# Novel Ubiquitin-derived High Affinity Binding Proteins with Tumor Targeting Properties*

**DOI:** 10.1074/jbc.M113.519884

**Published:** 2014-01-28

**Authors:** Susan Lorey, Erik Fiedler, Anja Kunert, Jörg Nerkamp, Christian Lange, Markus Fiedler, Eva Bosse-Doenecke, Maren Meysing, Manja Gloser, Chris Rundfeldt, Una Rauchhaus, Ilka Hänssgen, Thomas Göttler, Arnd Steuernagel, Ulrike Fiedler, Ulrich Haupts

**Affiliations:** From Scil Proteins GmbH, Heinrich-Damerow-Strasse 1, 06120 Halle (Saale), Germany

**Keywords:** Phage Display, Protein Engineering, Scaffold Proteins, Tumor Marker, Ubiquitin, Affilin, Biodistribution, Half-life Extension

## Abstract

Targeting effector molecules to tumor cells is a promising mode of action for cancer therapy and diagnostics. Binding proteins with high affinity and specificity for a tumor target that carry effector molecules such as toxins, cytokines, or radiolabels to their intended site of action are required for these applications. In order to yield high tumor accumulation while maintaining low levels in healthy tissues and blood, the half-life of such conjugates needs to be in an optimal range. Scaffold-based binding molecules are small proteins with high affinity and short systemic circulation. Due to their low molecular complexity, they are well suited for combination with effector molecules as well as half-life extension technologies yielding therapeutics with half-lives adapted to the specific therapy. We have identified ubiquitin as an ideal scaffold protein due to its outstanding biophysical and biochemical properties. Based on a dimeric ubiquitin library, high affinity and specific binding molecules, so-called Affilin® molecules, have been selected against the extradomain B of fibronectin, a target almost exclusively expressed in tumor tissues. Extradomain B-binding molecules feature high thermal and serum stability as well as strong *in vitro* target binding and *in vivo* tumor accumulation. Application of several half-life extension technologies results in molecules of largely unaffected affinity but significantly prolonged *in vivo* half-life and tumor retention. Our results demonstrate the utility of ubiquitin as a scaffold for the generation of high affinity binders in a modular fashion, which can be combined with effector molecules and half-life extension technologies.

## Teaser

**Background:** Targeting molecules to tumor cells is a promising mode of action for cancer therapy.

**Results:** Ubiquitin-based high affinity, specific, and stable binding molecules for extradomain B are accumulated in the tumor.

**Conclusion:** Ubiquitin may be engineered for high affinity target binding and modified with half-life extension technologies.

**Significance:** Ubiquitin qualifies as a well suited scaffold protein adaptable to specific tasks.

## Introduction

Monoclonal antibodies represent the best characterized class of biopharmaceuticals with broad applications in diagnostic and therapy. To date, over 30 antibodies have been approved in the United States and Europe for treatment of immunological and neoplastic diseases (1). Despite this track record, therapeutic antibodies have some restrictions mainly due to their complex structure and size (2). The complexity of antibodies based on their multimeric character, numerous disulfide bonds, and posttranslational modifications limits their utility for multispecific and targeting approaches. In addition, due to the large size, slow blood clearance, and prolonged retention time also in non-tumor tissues, antibodies are less suited for therapeutic areas such as tumor imaging or radioimmunotherapy, which require high tumor/blood ratios. Scaffold-based technologies may be applied to overcome these limitations. These technologies are focused on genetic engineering of small and less complex proteins of human or non-human origin to yield optimal properties regarding target binding, stability, modularity, and manufacturability in microorganisms. Due to the superior thermodynamic stability of scaffold proteins, multiple amino acid substitutions creating a target binding area of sufficient size are generally well tolerated without significant perturbation of the protein fold. Using this approach, proteins with high affinity and specificity against many different targets have been generated (3–5). Several scaffolds, such as Affibody®, Adnectin, and DARPin molecules, are currently under clinical evaluation (6–8). Thus, compared with antibodies, the hallmark of scaffolds consists in a reduced complexity at all levels from structure, genetic manipulation, biochemical handling through to GMP production, and analytics combined with excellent biophysical properties and ease of modular molecule designs.

We identified ubiquitin as an ideal scaffold protein for the generation of target-binding molecules. Ubiquitin is a small protein consisting of a single polypeptide chain of 76 amino acids folded in a highly compact α/β structure (9). Its outstanding biochemical properties (*i.e.* high solubility, temperature, pH, and proteolytic stability) make it well suited for genetic engineering (10). Ubiquitin is ubiquitously expressed in eukaryotes. It is mainly known as a cytosolic protein involved in numerous intracellular regulatory processes (11–13). However, ubiquitin also circulates in the blood of healthy humans in significant concentrations of approximately 100 ng/ml (14). Both extracellular and intracellular ubiquitin pools contain monomeric and multimeric proteins comprising a high diversity of differentially branched or linear polyubiquitin chains of variable length (15, 16).

Ubiquitin is reported to act as agonist of the CX chemokine receptor 4 with an affinity in the medium nanomolar range (17–19). However, the biological function of extracellular ubiquitin remains to be determined. From preclinical studies, some evidence has been presented that extracellular ubiquitin may function as an endogenous immune modulator with anti-inflammatory properties in trauma models but not in healthy animals (20–22). Furthermore, exogenous bolus applications of ubiquitin to healthy animals neither induced treatment-related toxicological effects nor influenced immunological functions or hematological parameters (23, 24). Therefore, the proposed immune modulatory function of systemic ubiquitin still needs to be clarified. In addition, strongly varying levels of ubiquitin in serum of healthy persons as well as patients (20) suggest that systemic ubiquitin is highly dynamic. Based on these considerations, a certain tolerability of administered ubiquitin and, potentially, of its derivatives is expected.

Extradomain B (ED-B)^2^ is a domain of an oncofetal fibronectin isoform inserted between the Fn^III^7 and Fn^III^8 domains as a result of alternative splicing (25). ED-B is normally absent in almost all adult tissues but is specifically expressed during wound healing and inflammation and in most cancer entities (26). Hence, ED-B has been considered as a promising cancer target for selective labeling of tumor vasculature and stroma, either for imaging purposes or for tumor-targeted treatment. Several approaches to identify and characterize ED-B-binding molecules for cancer therapy or diagnostics have been described, utilizing both antibody and scaffold technologies (27–30). Among these, the single chain antibody fragment (scFv) L19 is the most advanced molecule, with high binding affinity and specificity (30). Consequently, L19 represents the basis for the generation of a number of derivatives based on dimerization, fusion with cytokines, or conjugation with radiolabels (31–33). Clinical evaluation of the L19-derived molecules L19-TNFα, L19-IL2, and ^131^I-L19-SIP is still ongoing (34–36).

Here we describe the use of oncofetal fibronectin extradomain B as a target for the selection of ubiquitin-based binding proteins, a new class of high affinity and specific binding molecules. Based on a dimeric ubiquitin library, phage display selections followed by maturation approaches have been performed. Identified variants provide excellent results in thermodynamic stability as well as *in vitro* target binding and selectivity for the ED-B domain. Specific tumor accumulation was confirmed in quantitative biodistribution experiments. Half-life extension of the identified binding proteins significantly prolonged their retention time in the circulation and the tumor.

## EXPERIMENTAL PROCEDURES

### 

#### 

##### Construction of Library Modules

Human ubiquitin was used as a scaffold for library generation. Within the ubiquitin sequence, F45W, G75A, and G76A mutations were introduced by site-directed mutagenesis (QuikChangeII site-directed mutagenesis kit, Agilent Technologies, Santa Clara, CA). Based on *in silico* algorithms of the ubiquitin monomer calculating the stability effects of amino acid substitutions (37) and focusing on surface-exposed residues, nine amino acid positions were identified for randomization. A library constructed via linkage of two ubiquitin monomers was used for the selection of ED-B-binding molecules. The monomeric library modules SPW randomized at amino acid positions 2, 4, 6, and 62–66 and SPF randomized at amino acid positions 6′, 8′, and 62′–66′ were synthesized by Morphosys Slonomics (Martinsried, Germany) using a mixture of 19-amino acid coding premade double-stranded triplets excluding cysteine.

##### Phage Display Library Construction

Gene fragments of both library modules were used as template for a PCR introducing recognition sites for SfiI and EcoRI at the SPW module and MfeI and SfiI restriction sites at the SPF module, respectively. After digestion of the amplified fragments with EcoRI or MfeI, both fragments were ligated, yielding the linear SPWF library. The phagemid pCD87SA was provided by M. Paschke (38). The SPWF library and the phagemid were digested with SfiI and subsequently ligated. Aliquots of the ligation mixture were used for electroporation of *Escherichia coli* ElectroTen Blue cells (Agilent Technologies, Santa Clara, CA). Single colony PCR and sequence analysis of transformed bacteria were carried out to check for inserts of the correct size and sequence. Of all transformed clones, phagemid was purified using the QIAfilter® Plasmid Maxi Kit (Qiagen, Hilden, Germany). Thereafter, competent ER2738 cells (Lucigene, Middleton, WI) were electroporated with the phagemid library.

##### Phage Display Selection and Screening of Variants

Preparation of phage particles displaying variants of SPWF ubiquitin library was performed as described (39). A total of four panning rounds were conducted. For selection, N-terminal biotinylated 67B89 target protein (Nb67B89) was immobilized on Reacti-Bind Neutravidin strips (Pierce). A phage suspension of >10^12^ phages was added to the well of target-coated and preblocked Neutravidin strips and incubated in the presence of a 10-fold molar excess of 6789 off-target. Wells were washed with PBS containing 0.1% Tween 20 (PBST). Elution of bound phages was performed via pH shift achieved by incubation with a 200 mm glycine buffer adjusted to pH 2.2. For selection in solution, performed in the third and fourth round of panning, phage suspension was incubated with Nb67B89 in the presence of a 10-fold excess of 6789 off-target, followed by pull-down with Streptavidin beads (Invitrogen). Selection pressure was increased over the rounds via increasing washing stringency, reducing Nb67B89 target concentration and eluting via competition with an excess of 67B89 target.

For hit screening of selected binding molecules, phagemids were prepared from *E. coli* culture, and SPWF inserts were subcloned into pPR-IBA1 expression vector (IBA-Solutions for Life Science, Göttingen, Germany), followed by transformation of electrocompetent NovaBlue (DE3) cells (Agilent Technologies, Santa Clara, CA). Single colonies were picked, and protein was expressed on a small scale. Cell pellets were resuspended in PBST and lysed in the presence of lysozyme via 3-fold freeze thaw cycles. Hit-ELISA was performed on a Biomek FX (Beckman Coulter, Krefeld, Germany). A 10-fold dilution of the lysates in PBS was incubated with 67B89 and 6789 proteins coated on the wells of 96-well plates (MediSorp^TM^, Nalgene Nunc International, Penfield, NY). After washing with PBST, bound molecules were detected via anti-Ubi-Fab-POD (AbD Serotec, Oxfordshire, UK). Variants having a target/off-target binding of >2 and 100% sequence functionality were defined as hits.

##### Maturation and Screening of Binding Molecules

Ribosome display selection was used for affinity maturation. The SPW module of variant 19270, identified in phage display selection, was recombined with the SPF library module. Functional elements necessary for ribosome display selection were attached at the 5′- and 3′-ends of the library using standard cloning procedures. A total of four ribosome display cycles were performed (40). *In vitro* transcription and translation were realized using the PureExpress® *in vitro* protein synthesis kit (New England Biolabs, Ipswich, MA). For selection in solution, ternary complexes were incubated with Nb67B89 in PBSMT (PBS supplemented with 30 mm magnesium acetate and 0.05% Tween 20) in the presence of a 10-fold molar excess of 6789. Target-bound ternary complexes were recovered by incubation with Streptavidin beads. Selection of high affinity and specific binding molecules was supported by several washings with PBSMT. mRNA obtained after resolution of ternary complexes was reverse transcribed. The derived library was reamplified in two PCRs, restoring the functional modules necessary for the next ribosome display cycle. Selection pressure over all ribosome display cycles was increased by reducing the duration of target incubation and increasing washing stringency. After the fourth round, enriched binding molecules were subcloned and expressed as described above. Single colonies were analyzed for target/off-target binding in a lysate-based hit-ELISA (see above) with or without preincubation of lysates in mouse serum. Variants having a target/off-target binding ratio of >10, preservation of binding activity after serum incubation of >70, and 100% sequence functionality were defined as hits.

Based on variant 46877, another maturation was performed with the aim of increasing thermal stability and solubility. All variable positions were separately rerandomized by PCR using primer pairs containing an NNK triplet at the indicated single amino acid position. The obtained pool of variants was directly subcloned into pPR-IBA1 and transferred into electrocompetent NovaBlue (DE3) cells. Single colonies were picked, and protein was expressed and lysed as described above. For identification of heat-stable variants, lysates were incubated for 2 h at 50 °C before ELISA on target and off-target was performed. Protein expression was determined from lysate fractions by SDS-PAGE using NuPAGE Novex 4–12% BisTris gels (Invitrogen). To further increase expression yield and stability, an F63P exchange was introduced by site-directed mutagenesis into variants 65137, 65347, and 65351, identified in ribosome display maturation.

##### Target Expression, Purification, and Biotinylation

Based on the published DNA sequence (Uniprot ID P02751, isoform 7, sequence 1080–1541), genes for ED-B containing 67B89 and off-target 6789 proteins were obtained via gene synthesis (Geneart, Regensburg, Germany). For production of tag-free proteins, 67B89 and 6789 were cloned into pET28a expression vector and subsequently transferred into electrocompetent HMS174 (DE3) cells (Merck4Biosciences, Darmstadt, Germany). Following protein expression and cell harvest, the cell pellet was solubilized, and the protein was purified via a Q-Sepharose FF column, ammonium sulfate precipitation, and a Phenyl HP and Q Sepharose HP column. All chromatographic purification steps were carried out on Äkta chromatography systems (GE Healthcare).

For production of 67B89thc and 6789thc proteins, target and off-target genes were subcloned into pET20b vector (Merck4Biosciences), followed by C-terminal insertion of a G_4_SCP sequence. After transformation of NovaBlue (DE3) cells and protein expression, cell pellets were lysed, and protein in the supernatant was purified via immobilized metal affinity chromatography on a nickel-nitrilotriacetic acid column (5 ml; Qiagen, Hilden, Germany) and on a Superdex 75 16/600 column.

For a preferentially N-terminal biotinylation, 67B89 protein was dialyzed against 50 mm phosphate buffer, pH 6.5. A 10-fold molar excess of the EZ-Link Sulfo-NHS-LC-Biotin biotinylation reagent (Pierce) was added to 200 μl of a 1.4 mg/ml protein solution, followed by incubation for 24 h at 4 °C. Subsequently, 120 volume of 1 m Tris/HCl, pH 7.5, was added, and the final solution was dialyzed against PBS.

##### Binding Protein Expression and Purification

Binding molecules subcloned into pPR-IBA1 were expressed as proteins with C-terminal Strep-tag in NovaBlue (DE3) cells on a 1-liter scale. After cell lysis and lysate clarification, the supernatant was purified via a StrepTactin Super Flow column (IBA-Solutions for Life Science) according to the manufacturer's instructions. Subsequent size exclusion chromatography was realized in PBS, pH 7.4, on a Superdex 75 pg 16/600 column.

For the production of tag-free binding protein 77405, the coding DNA for 77405 was subcloned into pET-Sumoadapt vector (42) and subsequently transferred into electrocompetent NovaBlue (DE3) cells. Following expression, the binding protein was purified from the cell lysate via immobilized metal affinity chromatography. The His_6_-SUMO tag was cleaved off using SUMO hydrolase (43) and separated by immobilized metal affinity chromatography. The flow-through fraction, containing 77405 protein, was concentrated and loaded onto a Superdex 75 pg 26/600 column equilibrated in PBS. Tag-free NCP3, the corresponding wild-type ubiquitin dimer protein, was subcloned into pET20b and expressed in NovaBlue (DE3) cells as described. Protein was purified on SP Sepharose FF 16/100, Superdex 75 pg 50/900, and Q Sepharose FF 26/130.

For specificity testing via affinity chromatography, 77405 was genetically N-terminally fused to wild-type ubiquitin carrying an S57C substitution via standard cloning procedures. The coding DNA of the obtained 92787 variant was subcloned into pET20b, and protein was expressed in NovaBlue (DE3). After cell lysis and lysate clarification, 92787 protein was purified on an SP Sepharose HP and a Superdex 75 pg column.

For preparation of 77405-Fc, the coding DNA sequence of 77405 was ligated to the DNA sequence of IgG1-Fc fragment (Uniprot ID P01857, sequence 104–329). Following gene synthesis and subcloning into an appropriate expression vector, protein was obtained by expression in a eukaryotic expression system and subsequent purification (Sino Biologicals, Beijing, China). For production of 77405-MSA, the coding DNA fragment for 77405 was ligated to the DNA sequence of mouse serum albumin, yielding an N-terminal MSA fusion of 77405. A *Hansenula polymorpha* strain for secretory expression of 77405-MSA was generated by Artes Biotechnology GmbH (Langenfeld, Germany). Protein production and purification was carried out by Blirt (Gdansk, Poland).

##### Protein Modification

For PEGylation, 77405 protein was incubated with a 10-fold molar excess of a 40 kDa-branched PEG-aldehyde (Jenkem Technology USA Inc., Allen, TX) in 0.1 m sodium acetate, pH 5.5, for 2.5 h at 25 °C, followed by subsequent reduction. After termination of the reaction, mono-PEGylated 77405 protein was isolated on an SP Sepharose HP 26/160 column followed by a Superdex 200 pg 26/600 column.

##### Analytical Size Exclusion Chromatography

Analytical size exclusion chromatography was carried out on Superdex 200 and Superdex 75 Tricorn 10/300 columns (GE Healthcare), using an Ultimate 3000 SD chromatographic system (Dionex GmbH, Idstein, Germany). For molecular size determination by multiangle laser light scattering (MALS), a MiniDawn TREOS on-line MALS detector and a tREX refractive index detector (Wyatt Technology Europe GmbH, Dernbach, Germany) were coupled to the chromatographic system.

##### Surface Plasmon Resonance

Surface plasmon resonance measured on a Biacore^TM^ 3000 system (GE Healthcare) was used for analysis of binding of ubiquitin-based molecules to fibronectin extradomain B. Target and off-target proteins 67B89thc and 6789thc were covalently coupled to CM5 chips via the C-terminal cysteine (GE Healthcare) according to the manufacturer's instructions. Different concentrations of the binding proteins in PBST in a range of 0–500 nm were analyzed for binding to 67B89thc and 6789thc immobilized in the control flow cell. Dissociation constants (*K_D_*) were calculated by applying a global kinetic fit model (1:1 Langmuir) using BIAevaluation software (Biacore^TM^). All traces were corrected by on-line subtraction of the signals obtained in the control flow cells.

##### ELISA and Radioimmunoassay

ELISAs and radioimmunoassays were carried out in 96-well microtiter plates (Medisorp^TM^, Nalgene Nunc International). Wells were coated with the target or corresponding off-target protein. The wells were washed, blocked, and washed again. Various concentrations of the variants were applied. After incubation for 1 h and several washing steps with PBST, anti-Ubi-Fab-POD was added. Enzymatic activity was determined based on cleavage of 3,3′,5,5′-tetramethylbenzidine substrate (Kem-En-Tec Diagnostics A/S, Copenhagen, Denmark). Absorbance at a wavelength of 450 nm was measured on a Sunrise ELISA reader (Tecan, Crailsheim, Germany). For determination of serum stability, a concentration range of binding proteins was incubated in 90% mouse serum for 1 h at 37 °C, followed by target incubation and subsequent reassessment of binding affinity by ELISA.

For radioimmunoassay measurements, target was incubated with radiolabeled molecules accordingly, and binding was determined via direct measurement of radioactivity with a γ-counter (Wallace Wizard 2470, PerkinElmer Life Sciences). Apparent affinity constants (*K_D_*) were determined by raw data fitting according to hyperbolic binding behavior (SigmaPlot version 12.0, Systat Software, Erkrath, Germany).

##### Thermal Transition

Thermal transition of intrinsic tryptophan fluorescence was measured in a FluoroMax-3 fluorimeter (Horiba Jobin Yvon, Unterhaching, Germany) with Peltier element LFI-3751 (Wavelength Electronics, Bozeman, MT) over a temperature range of 20–90 °C at a 1 °C/min increment/decrement. Binding proteins were tempered in a quartz cuvette at a 1 μm concentration in PBS, pH 7.4. Fluorescence was measured at 280 nm excitation and 352 nm emission (bandwidth, 5 nm). Raw data were plotted against temperature, and *T_m_* was depicted from the maximum of the first deviation of the fluorescence curve.

##### Preparation of Cell Lysates

Cell pellets of Wi38 (CCL-75, LGC Standards GmbH, Wesel, Germany), NHDF (C12302, Promocell, Heidelberg, Germany), and HT29 (ACC-299, DSMZ, Heidelberg, Germany) were resuspended in cell extraction buffer (Invitrogen), and cell lysates were prepared according to the suppliers' instructions for Western blot analysis.

The NHDF cell pellet used for lysate preparation for affinity chromatography was resuspended in PBS supplemented with 1 mm EDTA, 0.1% SDS, 10% glycerin, 1 mm Pefabloc (Sigma-Aldrich), and 1% Triton X-100. The suspension was incubated for 30 min at room temperature under gentle stirring, followed by ultrasonication and centrifugation. The supernatant was used for affinity chromatography experiments.

##### Affinity Chromatography on 92787 Immobilized Resin

All chromatographic steps were performed on an Äkta chromatography system. 10.5 mg of 92787 protein was coupled to 10 ml of SulfoLink Coupling Resin (Pierce) according to the manufacturer's instructions, resulting in a static binding capacity of 700 μg of 67B89/ml of resin. Columns containing a volume of 1 ml of resin were used for analytical affinity chromatography. 200 μg of 67B89 target protein, corresponding to an amount enabling 15% 92787 saturation on the column, were spiked into 2 ml of PBS, NHDF cell lysate, or mouse serum (Sigma-Aldrich) and loaded onto a column. After column washing using at least 20 column volumes of PBS, pH 7.4, 92787-bound 67B89 was eluted with 10 column volumes of elution buffer (PBS, pH 2.2). Fractions of 0.5 ml were collected, and samples of 15 μl each were analyzed by SDS-PAGE on NuPAGE Novex 4–12% BisTris gels.

##### Western Blot

60 μg of protein from the respective cell lysates; 0.5, 2, or 5 μg of cellular fibronectin (Calbiochem, Bad Soden, Germany); and 50 ng each of 67B89 and 6789 were subjected to SDS-PAGE on NuPAGE Novex 4–12% BisTris gels and transferred to PVDF membranes (Bio-Rad) by electrophoresis. Membranes were blocked overnight at 4 °C using 5% dry milk in PBST, followed by incubation with 10 nm 77405 protein for 1 h at room temperature. ED-B binding was detected after incubation with a rabbit anti-Strep-tag IgG (Genscript, Piscataway, NJ) and an anti-rabbit IgG POD antibody (Sigma-Aldrich). Fibronectin was detected on separate membranes using a monoclonal anti-human fibronectin (R&D Systems, Wiesbaden-Nordenstadt, Germany) and an anti-mouse IgG POD antibody (Sigma-Aldrich). Following incubation with the substrate ECL Western blot detection reagent (GE Healthcare) for 1 min, blots were developed by 1 min of exposition. Antibodies were stripped from the membranes using 0.2 m glycine buffer, pH 2.2, supplemented with 0.1% SDS and 1% Tween 20. β-Tubulin was detected using an anti-β-tubulin antibody (Sigma-Aldrich) and an anti-mouse IgG POD antibody (Sigma-Aldrich).

##### Cell Binding Experiments

For cell binding experiments, Wi38 cells with high ED-B expression and NHDF cells with low ED-B expression were used. 30,000 cells were seeded per well of 4-well chamber slides (Fisher). After cultivation, cells were washed and incubated with 10 nm Strep-tagged 77405 or NCP3 protein in culture medium for 1 h at 37 °C. Cells were washed and fixed, followed by blocking with 5% horse serum in PBS. ED-B binding was detected after incubation with a rabbit anti-Strep-tag IgG antibody (Genscript, Piscataway, NJ) and a goat anti-rabbit IgG Alexa 488 antibody (Life Technologies). Cells were counterstained with DAPI (Sigma-Aldrich) and embedded in Mowiol. Green fluorescence indicative of ED-B staining was detected on an Axia Scope A1 (Zeiss, Jena, Germany) using an EX BP 470/40, BS FT 495, and EM BP 525/50 filter. Cell nuclei were detected using the following filter: EX G 365, BS FT 395, EM BP 445/50.

##### Immunohistofluorescence

Immunohistofluorescence was performed on cryopreserved F9 slices of 6 μm thickness. Slices were washed with PBST, fixed, and blocked with 5% horse serum. Incubation with 77405 and NCP3 protein and detection of target binding was carried out analogously to the cell binding experiments. CD31 staining was performed by using a rat anti-mouse CD31 IgG2a antibody (ab56299, Abcam, Cambridge, UK) and a goat anti-rat IgG Alexa 594 antibody (A11007, Life Technologies). Cell nuclei were counterstained with DAPI. Blue and green fluorescence of nuclei and ED-B, respectively, were detected as described. Red fluorescence of CD31 was detected using the following filter: EX BP 546/12, BS FT 560, EM BP 575–640. Colocalization of ED-B and CD31 expression was assessed after merging both fluorescence images.

##### Radioiodination of Recombinant Proteins, Analytics, Biodistribution, and Pharmacokinetic Experiments

Radioiodination of recombinant proteins, analytics, biodistribution, and pharmacokinetic experiments were performed at Chelatec SAS (Saint-Herblain, France). NCP3 and 77405 proteins were radioiodinated directly via the IODO-GEN method (45). Alternative labeling was performed for 77405, 77405-Fc, 77405-PEG and 77405-MSA by indirect iodination using *N*-succinimidyl 3-^125^I-benzoate (^125^I-SIB) (46). The labeled proteins were purified by gel filtration (Sephadex G25, PD10) and eluted in PBS, pH 7.3. Purity was determined by TLC and analytical size exclusion chromatography with radiodetection. Target binding affinity and immunoreactivity were checked by a radioimmune assay and via linear extrapolation of binding at infinite antigen excess on 67B89 target and 6789 control-coated beads (47).

##### Cell Lines and Animals

Mouse embryonal teratocarcinoma cells (F9) were provided by Cell Line Services (400174; Eppelheim, Germany). Female 129S1/SvImj mice (8 weeks old) were obtained from the Jackson Laboratory (Bar Harbor, ME). Female CD1 mice were provided by Charles River (Lille, France). For tumor induction in 129S1/SvImj mice, 5 × 10^6^ F9 cells were subcutaneously injected into the right flank. Tumor growth was measured using a caliper. Tumor volume was calculated according to the formula, (*a* × *b*^2^)/2, where *a* and *b* are the dimensions of the tumor. Housing, treatment, and sacrifice of the animals were performed according to European Union Directive 2010/63/EU on the protection of animals used for scientific purposes.

##### Biodistribution

For biodistribution experiments, tumor-bearing 129S1/SvImj mice with an average tumor volume of 250 mm^3^ were injected into the tail vein with radioiodinated proteins at a dose of 11.5 nmol/kg, a volume of 5 ml/kg, and a specific activity of 0.2 or 0.4 mCi/kg. At the time of sacrifice, organs of interest and the tumor of three mice of each group were excised, weighed, and counted in a γ-counter. Blood was sampled at different time points and at sacrifice for serum preparation.

##### Pharmacokinetics

For pharmacokinetics, CD1 mice were treated with ^125^I-SIB-labeled proteins as described above for biodistribution experiments. A total of 10 bleeding time points were assessed over 24 and 96 h, respectively. At each bleeding time point, blood from the saphenous vein of three non-anesthetized mice was collected in Microvette® tubes with clotting activator (Sarstedt, Nümbrecht, Germany). Tubes were centrifuged for 5 min at 10,000 × *g* for serum preparation, and radioactivity was measured in a γ-counter.

## RESULTS

The introduction of a universal *de novo* binding site on the surface of ubiquitin was guided by *in silico* calculations on the stability of ubiquitin, considering the influence of amino acid substitutions in relation to their solvent accessibility. Based on the three-dimensional structure of human ubiquitin (Protein Data Bank entry 1ubi), eight amino acid positions (Gln-2, Phe-4, Lys-6, Gln-62, Lys-63, Glu-64, Ser-65, and Thr-66) were identified as tolerating a high degree of mutations without significantly destabilizing the ubiquitin structure itself. In addition, position Leu-8 was selected for its close proximity to this artificial binding paratope aiming at the enlargement of the binding interface. To facilitate spectrophotometric analysis, an additional F45W amino acid substitution was introduced into the sequence without affecting the overall protein stability (48). Two Gly/Ala substitutions in positions 75 and 76 resulted in improved manufacturability of dimeric ubiquitin due to resistance against enzymatic cleavage by *E. coli* deubiquitinases (49).

**FIGURE 1.**
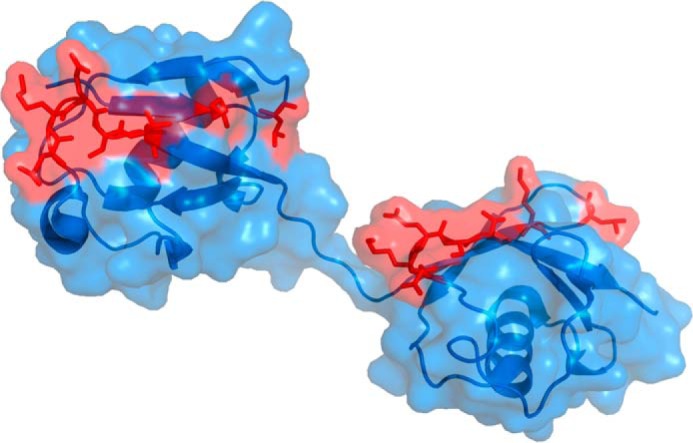
**Model of ubiquitin dimer used to design the ubiquitin scaffold.** The C-α trace is shown as a schematic diagram, and the accessible surface area is represented. Amino acid residues selected for generation of the ubiquitin library are *highlighted* in *red* with their original side chains. A structure based on Protein Data Bank entry 2W9N was generated using the program PyMOL.

Two ubiquitin monomer-related libraries, SPW and SPF, of theoretically 1.7 × 10^10^ and 8.9 × 10^8^ individual variants were synthesized. For both libraries, a continuous solvent-accessible surface area of ∼670 Å^2^ was calculated based on the wild-type ubiquitin sequence. DNA analysis of each library revealed 89 and 90% sequence integrity for SPW and SPF, respectively. By genetic head-to-tail fusion of both monomer library modules, an SPWF dimer library was obtained, with randomized positions at Gln-2, Phe-4, Lys-6, Gln-62, Lys-63, Glu-64, Ser-65, Thr-66, Lys-6′, Leu-8′, Gln-62′, Lys-63′, Glu-64′, Ser-65′, and Thr-66′ (Fig. 1 and Table 1), resulting in a theoretical complexity of ∼1.5 × 10^19^ possible combinations. Subcloning of the SPWF library in a phagemid vector and cell transformation yielded a total of 2.5 × 10^10^ clones. Sequence analysis revealed SPWF library inserts of preserved sequence integrity in 76% of the analyzed variants.

**TABLE 1:** **Characterization of ED-B binding of Strep-tagged binding molecules**

Selection	Variant	Randomized position															*K_D_*	*T_m_*	Stability (*K_D_* increase)*^a^*
		SPW								SPF									
		2	4	6	62	63	64	65	66	6′	8′	62′	63′	64′	65′	66′			
																	*nm*	°*C*	*-fold*
	Ubiquitin	Gln	Phe	Lys	Gln	Lys	Glu	Ser	Thr	Lys	Leu	Gln	Lys	Glu	Ser	Thr		82	
Phage display	19270	Thr	Trp	His	Asn	Phe	Lys	Leu	Ser	Ser	Phe	His	Tyr	Leu	Pro	Lys	14*^b^*	ND*^c^*	15
Maturation	46877	Thr	Trp	His	Asn	Phe	Lys	Leu	Ser	His	Gln	Gly	Trp	Gln	Ala	Pro	0.04*^d^*	54	1.9
	65347	Arg	Trp	His	Asn	Phe	Lys	Leu	Ser	His	Gln	Gly	Trp	Gln	Ala	Pro	0.03*^d^*	62	ND
	65351	Trp	Trp	His	Asn	Phe	Lys	Leu	Ser	His	Gln	Gly	Trp	Gln	Ala	Pro	0.05*^d^*	60	ND
	65137	Val	Trp	His	Asn	Phe	Lys	Leu	Ser	His	Gln	Gly	Trp	Gln	Ala	Pro	0.07*^d^*	62	ND
	77405	Arg	Trp	His	Asn	Pro	Lys	Leu	Ser	His	Gln	Gly	Trp	Gln	Ala	Pro	0.20*^d^*	66	ND
	92787	Arg	Trp	His	Asn	Pro	Lys	Leu	Ser	His	Gln	Gly	Trp	Gln	Ala	Pro	0.13*^d^*	60	1.8*^e^*

*^a^* Determined after incubation in mouse serum for 1 h at 37 °C by ELISA *versus* 67B89.

*^b^* Determined by ELISA measurement.

*^c^* ND, not determined.

*^d^* Determined by SPR measurement.

*^e^* Cysteine in 92787 protein was blocked by iodoacetamide.

### 

#### 

##### SPWF Library Selection of ED-B-binding Molecules

The SPWF library was used for the selection of ED-B-binding molecules. Pools from phage display selection rounds 3 and 4 were subcloned for *E. coli* expression and analyzed by ELISA on 67B89 target and 6789 off-target with cell lysates. Whereas hits identified in round 3 displayed a relatively high degree of diversity, hits identified in round 4 showed strong approximation to a consensus sequence of Thr-2, Trp-4, His-6, Asn-62, Phe-63, Lys-64, Leu-65, Ser-66, Ser-6′, Phe-8′, His-62′, Tyr-63′, Leu-64′, Pro-65′, and Lys-66′ (Table 1).

##### Affinity Maturation

**FIGURE 2.**
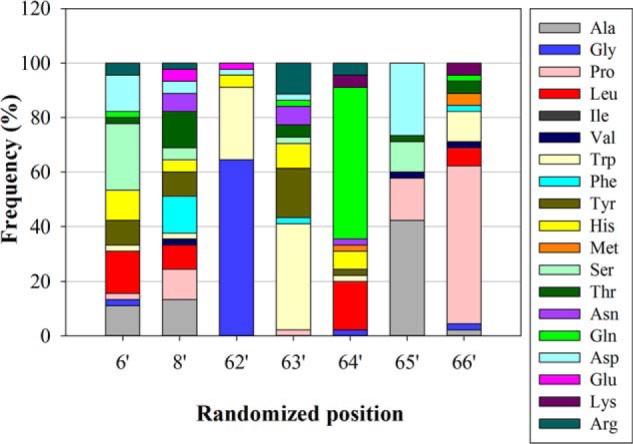
**Frequency of amino acids in randomized positions of 45 hits identified after maturation of variant 19270 by ribosome display.** The individual 19270 SPW module was recombined with an SPF library. Hits were analyzed for DNA sequence integrity. The frequency of every amino acid, except cysteine, is plotted *versus* the respective position of the SPF-binding module.

For affinity maturation of variant 19270, carrying the above mentioned consensus sequence, its individual N-terminal module was recombined with a library of the respective C-terminal module, similar to a procedure known as chain shuffling in antibody optimization. This resulted in a library covering a diversity of theoretical 8.9 × 10^8^ individual variants. Following ribosome display over four rounds of selection, hits were identified in a lysate-based ELISA on 67B89 target *versus* 6789 off-target with or without preincubation of the cell lysate in mouse serum. Sequence analysis of hits revealed approximation to a consensus sequence of Gly-62′, Trp-63′, Gln-64′, Ala-65′, and Pro-66′, representing the C-terminal binding motif of about 25% of the identified variants. In position 6′, serine was enriched in 25% of hits, whereas position 8′ did not display a relevant consensus (Fig. 2). Another maturation approach was used, aiming at the identification of molecules with improved thermal stability and protein expression. For that purpose, all variable positions of the SPWF library were rerandomized individually in variant 46877 obtained from ribosome display selection. In a subsequent ELISA, 30 hits were identified. Matured variants showed 3–6-fold increased protein expression compared with the parent 46877 variant. Amino acid substitutions mediating increased expression levels were primarily localized in position 2 but also in position 63. For combination of expression-enhancing effects, substitutions in position 2 and 63 were recombined, yielding variant 77405 with T2R and F63P substitutions and 10-fold increased expression level compared with 46877 (Table 1).

##### Binding Molecules Expressed as C-terminal Strep-tagged Proteins

Binding molecules were expressed as C-terminal Strep-tagged proteins in the cytoplasm of *E. coli*. Chromatographic purification yielded proteins of >96% purity as determined by SE-HPLC and reverse phase HPLC. A single protein band corresponding to a size of 19 kDa was detected by SDS-PAGE.

**FIGURE 3.**
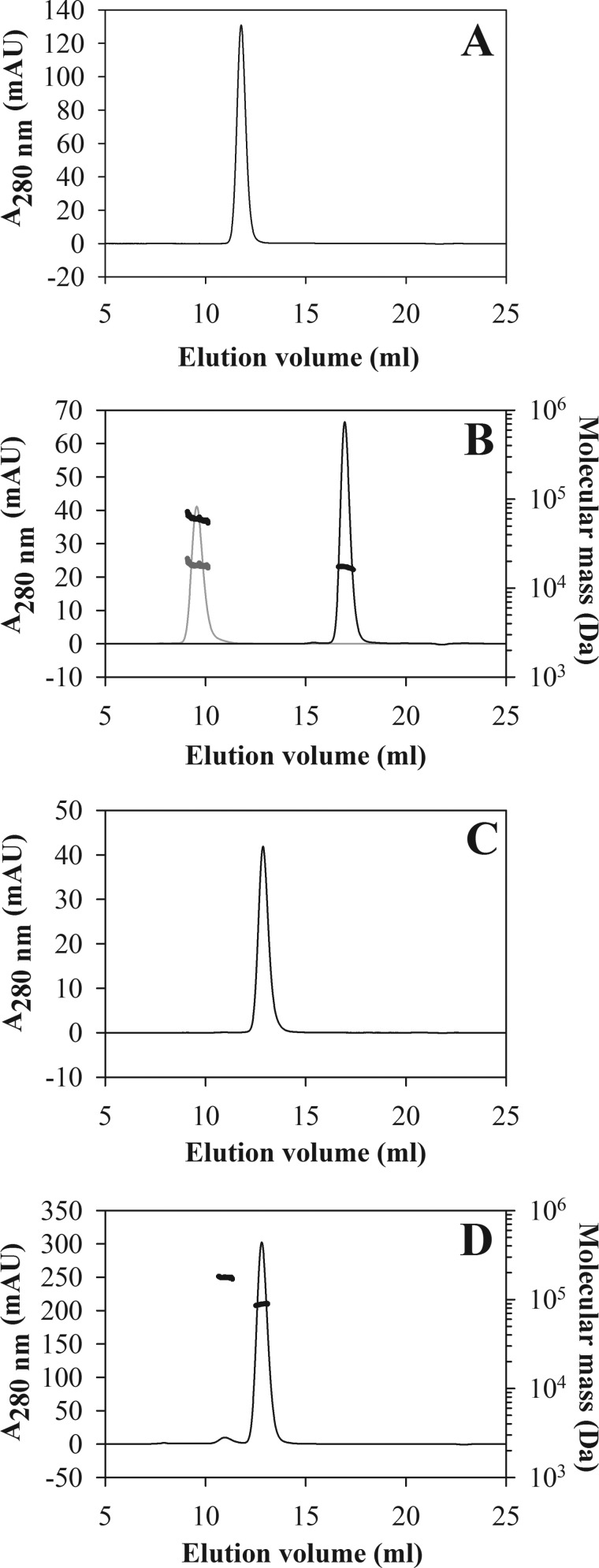
**Analytical size exclusion chromatography of NCP3, 77405 variant, and the 77405 conjugates 77405-PEG, 77405-MSA, and 77405-Fc.** Diubiquitin control protein NCP3 (80 μg) was applied to a Superdex 75 10/300 column (*A*). 77405 (30 μg; *black trace*) and 77405-PEG (22 μg of protein equivalent; *gray trace*) were applied to a Superdex 200 10/300 column (*B*). *Black dots* indicate molecular mass in the peaks as determined by MALS analysis; *gray dots* indicate molecular mass of the protein component alone in PEGylated 77405. 77405-MSA (33 μg) was applied to a Superdex 200 10/300 column (*C*). 77405-Fc (160 μg) was applied to a Superdex 200 10/300 column (*D*). *Black dots* indicate molecular mass in the peaks corresponding to the dimer and tetramer peaks, respectively. As running buffer, PBS (*A–C*) and 0.2 m sodium phosphate, 0.1 m arginine HCl, 10 mm NaCl, 0.1% 2-propanol, pH 6.5 (*D*) were used at a flow rate of 0.5 ml/min. *mAU*, milliabsorbance units.

The 77405 variant and the 92787 variant derived thereof as well as the corresponding wild-type ubiquitin dimer protein NCP3 were also produced as tag-free proteins for animal studies and affinity chromatographic analyses. Preparations of all proteins showed more than 95% purity according to reverse phase HPLC and SDS-PAGE. Analysis by SE-HPLC confirmed the monomeric character of the proteins with homogeneities of >99% (Fig. 3, *A* and *B*).

In order to evaluate options for modulation of plasma kinetics of binding molecules, purified 77405 protein was PEGylated with a 40 kDa-branched PEG moiety. PEGylated protein was obtained in an overall yield of 11%. Homogeneous mass distribution of the purified mono-PEGylated 77405 (77405-PEG) was confirmed by SE-HPLC/MALS, revealing a molecular mass of 60.1 kDa with a corresponding protein mass of 18.1 kDa (Fig. 3*B*).

77405 fused to mouse serum albumin (77405-MSA) was obtained with >98% purity, as judged by reverse phase HPLC and appeared >99% homogeneous on SE-HPLC (Fig. 3*C*).

A fusion of 77405 Affilin to the human IgG1 Fc domain (77405-Fc) was obtained as apparently pure protein as determined by SDS-PAGE. SE-HPLC/MALS analysis revealed 95% of the correctly assembled bivalent 77405-binding protein and 4.5% of apparently dimerized species with molecular masses of 88.6 and 177.3 kDa (Fig. 3*D*), corresponding to two and four individual chains, respectively. The endotoxin level of all preparations administered *in vivo* was determined to be below 20 endotoxin units/mg of protein.

##### Characterization of Target Binding

**FIGURE 4.**
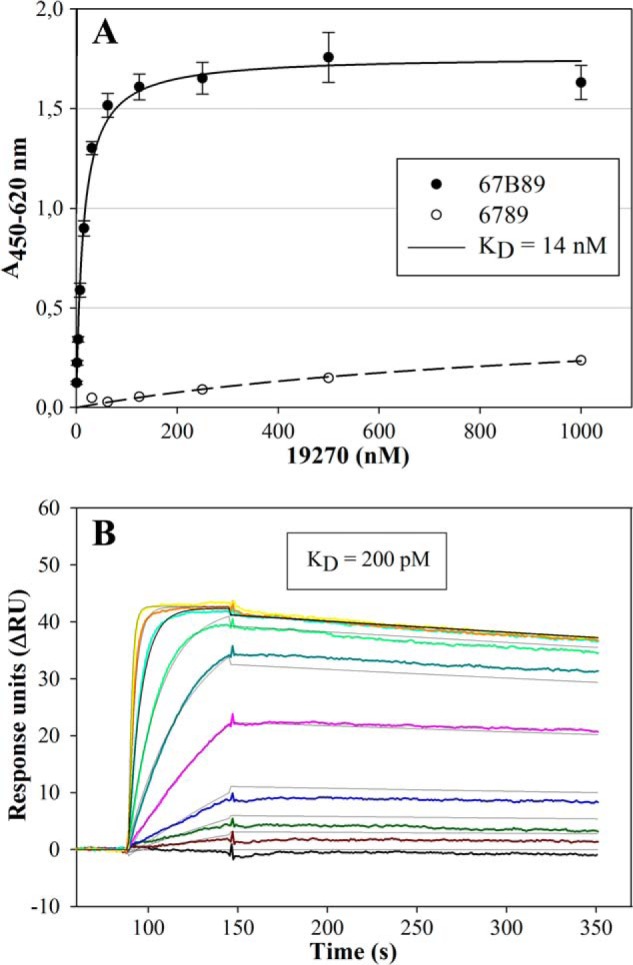
**Determination of the apparent dissociation constants of ED-B-binding molecules 19270 and 77405 by ELISA and SPR measurements, respectively.** *A*, 67B89 target and 6789 off-target were immobilized in the wells of a microtiter plate. Strep-tagged variant 19270 was applied in serial dilutions. Bound protein was detected by an anti-Ubi-Fab antibody fragment conjugated to POD. Absorption determined at 450 nm was plotted against protein concentration and subjected to hyperbolic curve fitting. The *K_D_* value is given in the *inset* and in Table 1. Target binding (*filled circle*) was determined in triplicates and is presented as a mean ± S.D. (*error bars*). Off-target binding (*open circle*) was determined as a single measurement. *B*, binding experiments of Strep-tagged 77405 on 67B89thc target and 6789thc off-target covalently coupled to CM5 chips. Different concentrations of the 77405 variant in a range of 0 (*black*) to 200 nm (*yellow*) were analyzed. As a running buffer, PBS containing 0.005% Tween 20 was used at a flow rate of 30 μl/min. Calculation of the dissociation constant (*K_D_*) was performed by applying a global kinetic fit model (1:1 Langmuir). All traces were corrected by on-line subtraction of the signals obtained in the 6789thc-immobilized control flow cells. The *K_D_* value is given in the *inset* and in Table 1.

Characterization of target binding of hits identified in phage display yielded binding affinities between 14 and 414 nm as determined by ELISA. Variant 19270 was characterized by an apparent dissociation constant (*K_D_*) of 14 nm and low nonspecific binding to 6789 off-target (Fig. 4*A*). However, binding affinity was about 15-fold diminished after incubation of 19270 in the presence of 90% mouse serum (Table 1).

Variant 19270-based affinity-matured molecules selected by ribosome display and screened for increased serum stability were analyzed for 67B89 target binding by SPR measurements, revealing dissociation constants between 30 and 616 pm. Variant 46877 showed an improved binding affinity toward 67B89 with a *K_D_* of 30 pm. ELISA performed after incubation of 46877 in the presence of 90% mouse serum revealed preservation of target binding (Table 1).

SPR-based binding analyses of matured variants, obtained after rerandomization of single amino acids of 46877 variant, yielded affinities between 30 and 200 pm and very slow off-rates in the order of 3 × 10^−6^ s^−1^ and on-rates in the order of 1 × 10^4^ m^−1^ s^−1^ (Fig. 4*B*). Overall, SPR measurements revealed a 1:1 binding mechanism.

Tag-free variant 77405 showed a binding affinity of 190 pm determined by SPR and 360 pm determined by ELISA measurements. Protein 92787, representing variant 77405 N-terminally fused to ubiquitin, was characterized by maintained binding affinity (*K_D_* = 130 pm). 77405 dimerization via Fc fusion resulted in a protein showing a 4-fold improved apparent binding affinity toward 67B89. Binding analysis of 77405-PEG and 77405-MSA provided apparent *K_D_* values that correspond to an ∼4-fold and 2-fold increase compared with 77405 (Table 2). SPR analysis of 77405-PEG and 77405-MSA indicated that association rate constants for ED-B binding were reduced by an order of magnitude compared with 77405, whereas dissociation rate constants remained largely unchanged (data not shown).

**TABLE 2:** **Binding analysis and protein purity of non-labeled and radiolabeled tag-free NCP3, 77405, and 77405-based proteins**

	Non-labeled		Labeled		
	*K_D_*	Purity	*K_D_**^a^*	Immunoreactivity	Purity*^b^*
	*nm*	%	*nm*	%	%
NCP3*^c^*		99			99
77405*^c^*	0.19*^d^*	100	0.29	93	91
NCP3*^e^*		99			99
77405*^e^*	0.36*^f^*	100	0.44	105	92
77405-Fc*^e^*	0.08*^f^*	95	1.37	98	91
77405-PEG*^e^*	1.32*^f^*	100	0.96	102	96
77405-MSA*^e^*	0.58*^f^*	99	ND*^g^*	ND	ND

*^a^* Determined by radioimmunoassay measurement on 67B89.

*^b^* Determined by SE-HPLC with radiodetection.

*^c^* Protein directly iodinated on tyrosine side chains.

*^d^* Determined by SPR measurement on 67B89thc.

*^e^* Protein indirectly iodinated on lysine side chains.

*^f^* Determined by ELISA measurement on 67B89.

*^g^* ND, not determined.

##### Target Binding Specificity

**FIGURE 5.**
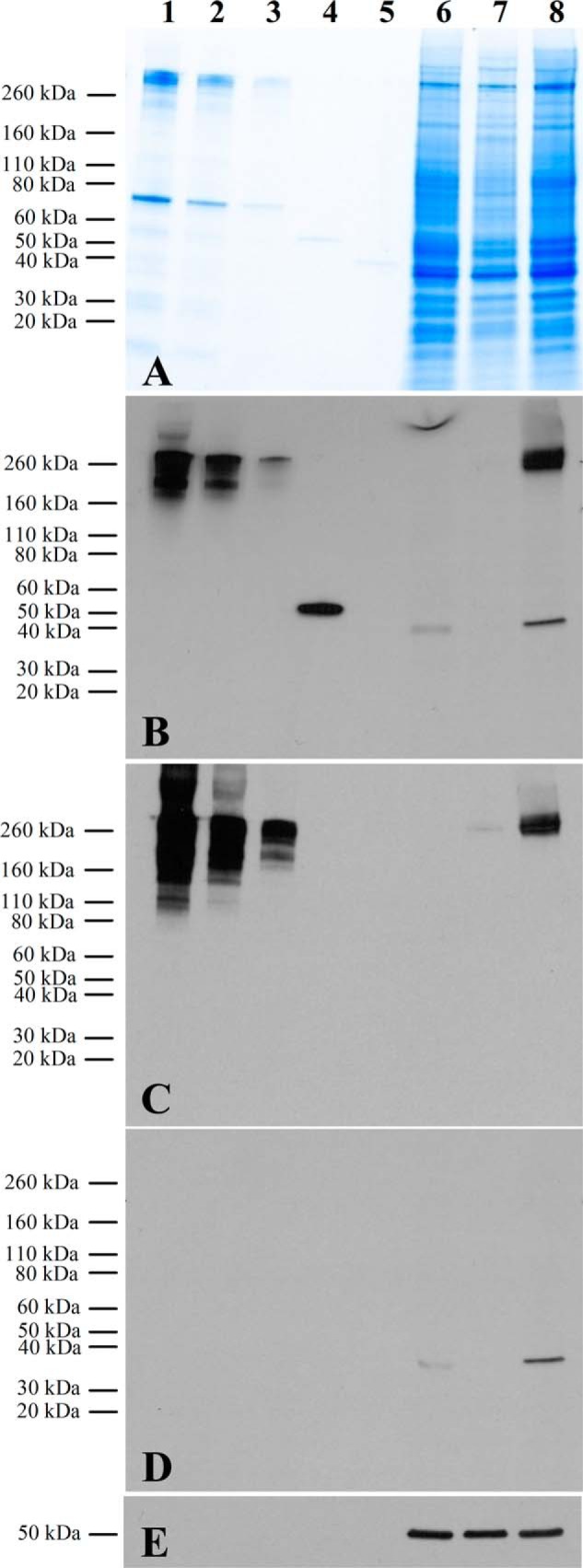
**Target binding specificity of 77405 as determined by Western blot analyses.** *A*, SDS-PAGE of analyzed samples. *B*, detection of ED-B target using the 77405 variant, a rabbit anti-Strep-tag, and an anti-rabbit POD-conjugated antibody. *C*, detection of fibronectin using a mouse anti-human fibronectin and an anti-mouse POD-conjugated antibody. *D*, control blot using the secondary anti-rabbit antibody yielding a single band at 40 kDa due to nonspecific binding of the secondary antibody. *E*, detection of β-tubulin using an anti-tubulin antibody. For all figures, *lanes* were loaded with the following samples: 5 μg of cellular fibronectin (*lane 1*), 2 μg of cellular fibronectin (*lane 2*), 0.5 μg of cellular fibronectin (*lane 3*), 50 ng of 67B89 (*lane 4*), 50 ng of 6789 (*lane 5*), 60 μg of protein from HT29 cell lysate (*lane 6*), 60 μg of protein from NHDF cell lysate (*lane 7*), and 60 μg of protein from Wi38 cell lysate (*lane 8*).

Target binding specificity of 77405 variant as determined by Western blot showed strong and specific detection of cellular ED-B-fibronectin in Wi38 cell lysate and isolated cellular fibronectin samples (Fig. 5*B*) corresponding to the fibronectin bands (Fig. 5*C*). NHDF cell lysate with low ED-B expression showed only a very slight ED-B band in the molecular weight range of cellular fibronectin. HT29 cell lysate without ED-B expression did not show any EDB-related bands (Fig. 5*B*). The lanes of HT29 and Wi38 cell lysates were characterized by a nonspecific band around 40 kDa size. This single band was related to nonspecific interaction of the secondary anti-rabbit antibody (Fig. 5*D*). There was no hint of nonspecific interactions of the Affilin with cellular proteins.

**FIGURE 6.**
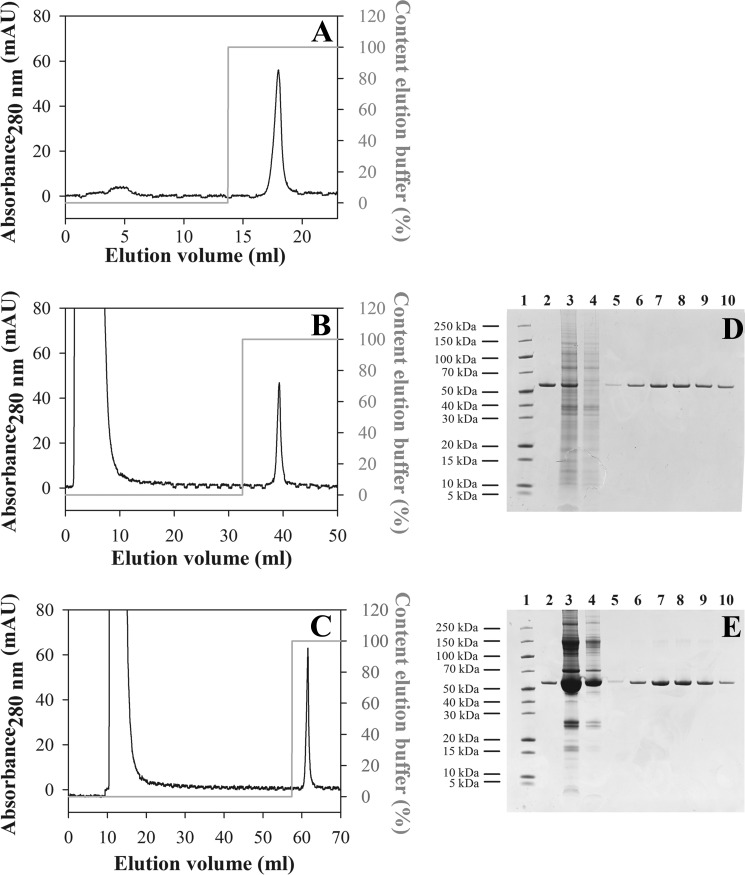
**Affinity chromatography on 92787-immobilized resin.** 200 μg of 67B89 target protein were added to 2 ml of PBS (*A*), NHDF cell lysate (*B*), or mouse serum (*C*) and loaded onto the column. After column washing, bound 67B89 was eluted with elution buffer (*gray line* represents the elution step). Fractions were collected and analyzed by SDS-PAGE. *D*, NHDF cell lysate. *Lane 1*, protein marker; *lane 2*, 1 μg of 67B89; *lane 3*, 10-μl column load; *lane 4*, 10-μl flow-through; *lane 5*, fraction 38.5 ml; *lane 6*, fraction 39 ml; *lane 7*, fraction 39.5 ml; *lane 8*, fraction 40 ml; *lane 9*, fraction 40.5 ml; *lane 10*, fraction 41 ml. *E*, mouse serum. *Lane 1*, protein marker; *lane 2*, 1 μg of 67B89; *lane 3*, 10 μl column load; *lane 4*, 10 μl flow-through; *lane 5*, fraction 60 ml; *lane 6*, fraction 60.5 ml; *lane 7*, fraction 61 ml; *lane 8*, fraction 61.5 ml; *lane 9*, fraction 62 ml; *lane 10*, fraction 62.5 ml. *mAU*, milliabsorbance units.

77405-related variant 92787 was immobilized in a site-directed manner on a chromatographic resin with a coupling yield of 51.2%. Binding capacity was determined at 0.7 mg of 67B89/ml of resin. NHDF cell lysate and human serum, each spiked with 200 μg of 67B89 and loaded onto the column, resulted in a specific elution peak (Fig. 6, *B* and *C*) corresponding to 67B89 protein eluted after application in PBS (Fig. 6*A*) without showing relevant contaminations, as shown in SDS-PAGE (Fig. 6, *D* and *E*). In all chromatographic runs, >85% of applied 67B89 protein was recovered.

##### Thermal Stability Measurements

Thermal stability measurements revealed a midpoint of thermal transition of 54 °C for variant 46877. Affinity maturation resulted in increased thermal stability with transition temperatures of between 60 and 66 °C (Table 1).

##### Cell-based Target Binding Experiments

**FIGURE 7.**
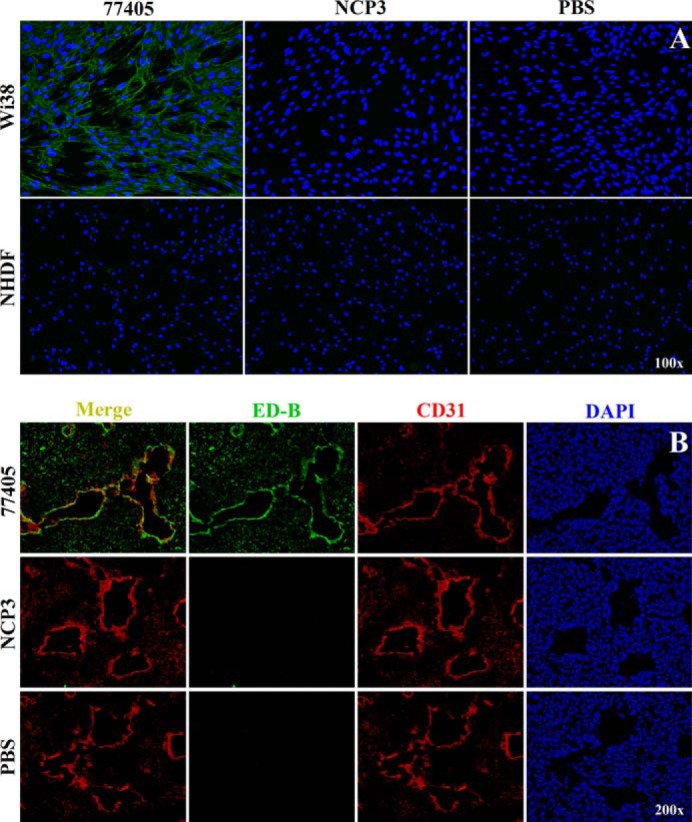
**Cell and tissue binding specificity of NCP3 and variant 77405.** *A*, cell binding on ED-B-positive Wi38 cells and NHDF cells with very low ED-B expression. Cells were seeded and cultured in 4-well chamber slides. For the staining procedure, cells were washed and incubated with 10 nm Strep-tagged 77405 or NCP3 followed by fixation. ED-B target binding was detected using an anti-Strep-tag IgG antibody and a goat anti-rabbit IgG Alexa 488 antibody. Cells were counterstained with DAPI. *Green fluorescence* reflects ED-B binding of proteins. *Blue fluorescence* is indicative of cell nucleus staining. *B*, immunohistochemical staining of F9 tumor slices. Cryopreserved F9 slices were fixed and blocked followed by incubation with 10 nm Strep-tagged 77405 and NCP3 control protein. Staining of bound proteins was carried out analogous to the cell experiments. Localization of vessels was detected with a rat anti-mouse CD31 IgG2a antibody and a goat anti-rat IgG Alexa 594 secondary antibody. Cells were counterstained with DAPI. *Blue* and *green fluorescence* are indicative of nuclei and ED-B, respectively. *Red fluorescence* of CD31 reflects vessel staining. Merging of ED-B- and CD31-stained images results in a *yellow* appearance indicative of vessel association of ED-B expression.

Cell-based target binding experiments using Strep-tagged 77405 showed a strong fluorescence indicative of ED-B expression on the matrix of Wi38 cells. In contrast, NHDF cells with low ED-B expression showed only a sparse decoration with 77405. No staining was observed with NCP3 on Wi38 or NHDF cells, respectively (Fig. 7*A*).

##### Immunohistochemistry

Immunohistochemistry performed on slices of cryopreserved F9 tumors with Strep-tagged 77405 and NCP3 proteins revealed strong fluorescence and almost exclusive vessel association of 77405 protein. NCP3 did not show any binding on F9 slices (Fig. 7*B*).

##### Radiolabeling Methods

Two radiolabeling methods have been chosen to investigate biodistribution and pharmacokinetics of variant 77405 and related proteins. In any case, labeling efficiency of the proteins was in a range between 40 and 70% with more than 97% radiopurity. Labeled proteins were more than 91% pure as determined by SE-HPLC. Immunoreactivity of all labeled proteins was higher than 93%. Binding activity toward 67B89 was retained for almost all binding proteins except for 77405-Fc, showing a 16-fold increase of *K_D_* value after labeling (Table 2). *In vitro* serum stability analysis of labeled proteins by TLC revealed no dehalogenation even after 16 h of incubation at 37 °C (data not shown).

**FIGURE 8.**
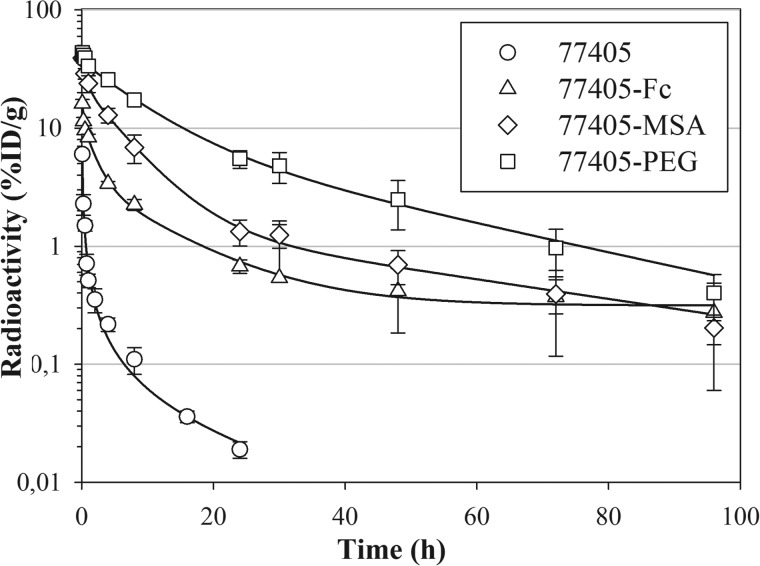
**Clearance of variant 77405 and 77405-based proteins 77405-Fc, 77405-PEG, and 77405-MSA from serum of healthy CD1 mice.** Proteins were labeled by indirect iodination using ^125^I-SIB and administered intravenously. At the indicated time points, animals were bled, and radioactivity was measured. Mean radioactivity (77405 (○), 77405-Fc (▵), 77405-PEG (□), and 77405-MSA (♢)) determined from *n* = 3 animals is plotted *versus* bleeding time points and specified by S.D. (*error bars*). Data on protein analytics and pharmacokinetics are summarized in Table 3.

Plasma pharmacokinetics of 77405 and modified 77405 molecules (*i.e.* 77405-PEG, 77405-MSA, and 77405-Fc) were evaluated following single intravenous dosing. The intravenous administration of radiolabeled proteins was well tolerated. Animals behaved normally and showed no signs of intolerance or toxicity, including local irritation. Pharmacokinetic evaluation of the serum level time course of labeled proteins in native CD1 mice revealed that all three half-life extension technologies resulted in significantly increased terminal half-lives of 77405-PEG, 77405-MSA, and 77405-Fc compared with the terminal serum half-life of 77405. In line with the prolonged half-life, the clearance of the radiolabel from serum was largely reduced for all three technologies, compared with unmodified 77405 (Table 3 and Fig. 8).

**TABLE 3:** **Pharmacokinetic parameters of ^125^I-SIB-labeled 77405 and 77405-related molecules determined in healthy CD1 mice**

	Mass	*T*½	AUC*^a^*	Clearance
	*kDa*	*h*	*(*μ*Ci/ml/h)*	*(ml/h/kg)*
77405	17.5	(6)*^b^*	0.4	935
77405-Fc	88.4	56	15.9	35
77405-PEG	60.3	20	63.0	7
77405-MSA	84.6	26	27.9	16

*^a^* Area under the curve.

*^b^* Value obtained from a three-exponential fit for the terminal half-life; however, this is at a level more than 2 orders of magnitude below the starting concentration, and data should thus be interpreted carefully.

##### Tissue Distribution Experiments

**FIGURE 9.**
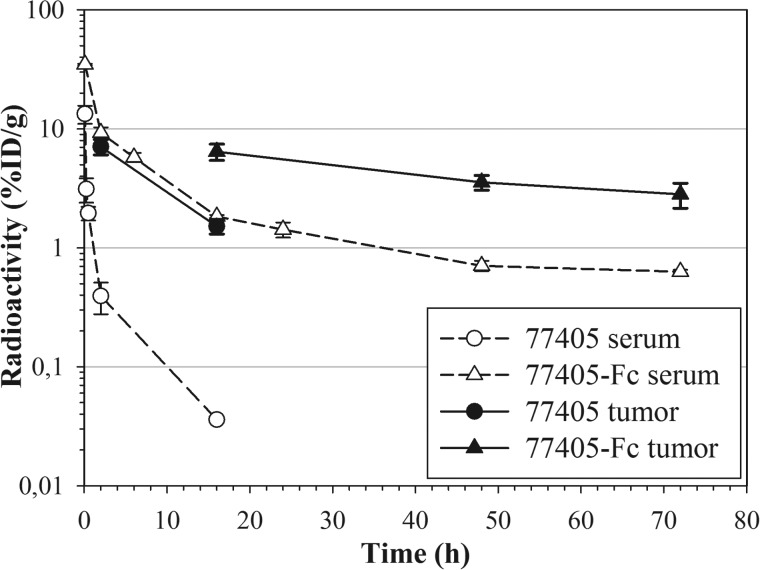
**Serum and tumor radioactivity of 77405 and 77405-Fc in F9 tumor-bearing 129S1/SvImj mice.** Proteins were labeled by indirect iodination using ^125^I-SIB and administered by intravenous injection. Tumor radioactivity was determined 2 and 16 h (77405, *filled circles*, *straight* l*i*ne) and 16, 48, and 72 h (77405-Fc, *filled triangle*, *straight line*) after administration from *n* = 3 animals. Serum radioactivity (77405 (*open circle*) and 77405-Fc (*open triangle*), *dotted lines*) was measured accordingly, including intermediate bleeding time points. Tumor and serum radioactivity are plotted *versus* time and presented as mean values ± S.D. (*error bars*).

Tissue distribution experiments in F9 tumor-bearing mice were initially performed with 77405 and NCP3 proteins labeled directly via tyrosine side chains. Although *in vitro* serum stability of protein-attached label was excellent, *in vivo* serum stability was diminished by dehalogenation, resulting in ∼50% free ^125^I detectable already 30 min after administration. For that reason, an alternative labeling approach, the indirect labeling of lysine side chains via ^125^I-benzoate, was established and showed strongly enhanced stability under *in vivo* conditions (data not shown). Biodistribution was performed in F9 tumor-bearing 129S1/SvImj mice intravenously injected with equimolar amounts of radiolabeled proteins. Two hours postinjection, radioactivity of blood and main organs was comparable for 77405 *versus* NCP3. At the 16 h time point, NCP3 had cleared from almost all organs, whereas 77405 showed an about 3–10-fold increased radioactivity in specified organs and blood compared with NCP3 (Table 4). However, overall radioactivity was determined on a very low absolute level. Tumor accumulation of 77405 protein was 2-fold increased *versus* NCP3 at 2 h and 15-fold increased at 16 h postinjection. The tumor/blood ratio of ^125^I-77405 protein increased from 1.8 at 2 h to 5.8 at 16 h after administration, respectively. For indirectly labeled ^125^I-SIB-77405, the absolute tumor accumulation remained comparable with that of the directly labeled 77405 protein (Fig. 9), but the tumor/blood ratio increased to 42 as seen 16 h postinjection.

**TABLE 4:** **Tumor accumulation and organ distribution of ^125^I-labeled NCP3 and 77405 variant in F9 tumor-bearing 129S1/SvImj mice given as % ID/g**

	Blood	Tumor	Liver	Kidneys	Heart	Lung	Spleen	Intestine
**^125^I-NCP3**								
2 h	3.85 ± 0.97	3.42 ± 0.76	1.45 ± 0.22	5.80 ± 1.35	1.24 ± 0.22	2.46 ± 0.11	2.38 ± 0.27	2.13 ± 0.05
16 h	0.04 ± 0.00	0.11 ± 0.00	0.06 ± 0.01	0.13 ± 0.01	0.03 ± 0.01	0.09 ± 0.02	0.07 ± 0.01	0.06 ± 0.01

**^125^I-77405**								
2 h	3.59 ± 0.36	6.61 ± 1.92	1.91 ± 0.14	3.96 ± 0.87	1.28 ± 0.15	3.38 ± 1.19	2.45 ± 0.52	3.30 ± 0.16
16 h	0.32 ± 0.08	1.83 ± 0.59	0.34 ± 0.06	0.36 ± 0.05	0.13 ± 0.02	0.52 ± 0.16	0.30 ± 0.05	0.67 ± 0.15

Increasing the serum half-life clearly resulted in prolonged tumor retention, as shown for 77405-Fc with 6.44% ID/g of tumor at 16 h postinjection and 2.82% ID/g of tumor detectable even 72 h after administration (Fig. 9). As expected due to the long half-life of the Fc fusion construct, the tumor/blood ratio of 77405-Fc was reduced to 3–5 compared with the tumor/blood ratio of unmodified 77405.

## DISCUSSION

Monoclonal antibodies are broadly applied in diagnostic and clinical applications. However, they share some limitations related to their molecular complexity and size and corresponding *in vivo* characteristics. Scaffold-based technologies may help to overcome some of these limitations by providing binding molecules with high affinity and specificity, which can be easily engineered in a modular fashion.

Ubiquitin is a protein of low molecular complexity and excellent stability. It can be produced in high yield in the cytoplasm of *E. coli*. Ubiquitin is 100% conserved among the mammals, which eliminates the need for species-specific surrogate molecules during preclinical development. In addition, ubiquitin is a systemic endogenous human protein that is expected to reduce the risk of potential immunogenicity because of the constant exposure of the human immune system to ubiquitin. Based on these advantageous properties, we choose ubiquitin as a scaffold molecule for the generation of *de novo* binding sites, forming the basis for the Affilin molecules. We have previously described the generation of a binding region on the surface of ubiquitin, demonstrating its general applicability as a scaffold (50). Based on the successful selection of target-specific binding variants derived from a single ubiquitin molecule, we now developed next generation binding molecules that are composed of two head-to-tail linked ubiquitin monomers. The resulting dimeric ubiquitin is stable in solution without forming aggregates at a concentration of up to 120 mg/ml.^3^ Furthermore, it shows high thermal stability with a midpoint of thermal transition around 82 °C. Modular composition of ubiquitin enables flexibility between the ubiquitin monomers (51, 52), thus allowing a certain degree of plasticity of an artificial binding paratope spanning both ubiquitins.

The amino acid positions to be randomized for the generation of a dimeric ubiquitin-based binding paratope were identified based on spatial considerations and *in silico* analyses. The substitutions in randomized positions were primarily introduced in β-sheets (Gln-2, Phe-4, Lys-6, Ser-65, and Thr-66) and β-turns (Leu-8, Gln-62, Lys-63, and Glu-64) of ubiquitin, representing relatively rigid secondary structure elements. The principle of introducing binding paratopes in secondary structure elements has been demonstrated for other scaffold proteins, such as Affibodies and DARPins. Affibodies, based on the bacterial staphylococcal protein A, are characterized by introduction of a binding paratope in α-helices (4). DARPins, as designed ankyrin repeat proteins, are artificially designed scaffold proteins with a binding paratope in β-turns and α-helices (53). For both scaffold approaches, introduction of binding sites in relatively rigid secondary structure elements has been discussed as being advantageous because of reduced entropy costs for structural rearrangements of binding domains upon target interaction that may occur in the case of flexible binding structures generated in hypervariable or single loops. Indeed, as presented for ubiquitin-based binding molecules, for Affibodies as well as for DARPins, specific and high affinity target interaction can be achieved with such rigid structures.

Based on the dimeric ubiquitin library, Affilin molecules were successfully selected against multiple targets, belonging to the groups of receptor-tyrosine kinases, cytokines, growth factors, cellular receptors, or matrix proteins (data not shown). Here we present results for the selection of binding molecules against ED-B, applying Tat-based phage display and ribosome display. The selection strategy aimed at first identifying an ED-B-binding molecule from a phage display library, followed by affinity maturation of initial hits via ribosome display. By combination of both selection methods in conjunction with a systematic screening strategy, we identified ED-B-binding molecules characterized by high target binding affinity and specificity as well as increased serum and thermal stability. Our findings show that randomization of the aforementioned positions in the library used for selection and maturation approaches did not significantly disturb the protein stability of the identified variants. Therefore, a high predictivity of the *in silico* calculations for the ubiquitin structure is indicated.

The library based on dimeric ubiquitin is characterized by a *de novo* binding site consisting of 15 randomized amino acid positions, which provide a sufficiently large paratope to facilitate picomolar affinities. Indeed, the matured variants display affinities in the range between 30 and 200 pm. Due to the dimeric composition of the binding molecules and the flexibility between the ubiquitin modules, one could expect a bivalent binding mechanism mediated by separate binding events of the ubiquitin monomer modules on the target. However, analysis of target binding behavior via SPR measurements yields responses that can be well fitted based on a 1:1 binding model. In addition, target binding analysis of the monomer modules of high affinity binders results in significant affinity loss of both monomers compared with the dimer Affilin (data not shown). These data suggest that the binding paratope is composed of both ubiquitin modules binding in a cooperative manner and behaving as a single binding interface.

Thermal stability of the selected variants was characterized by a midpoint of thermal transition between 60 and 66 °C. Although these data represent a reduced thermal stability compared with the stability of the wild-type ubiquitin dimer, it is still in an acceptable range for biotherapeutic applications. Crystal structure analyses of binders alone and in complex with the ED-B target^4^ demonstrate that the C-α trace of the Affilin is only marginally perturbed compared with the backbone of wild-type ubiquitin supporting maintenance of the overall structure. However, introduction of non-wild-type amino acids at the library positions influences the thermodynamic behavior of the molecule. Improved temperature stability can be forced by applying additional selection pressure during the selection and screening process.

To date, three other ED-B-binding proteins have been published. Two of them, a Fynomer® and an anticalin, are scaffold-based monomeric binding proteins obtained after primary phage display selection with binding affinities toward ED-B target in the low to medium nanomolar range (28, 29). A third protein, L19, is an scFv representing the most advanced ED-B-binding protein obtained after affinity maturation. Depending on the analytical method used, binding affinities between 872 and 54 pm were determined for L19 (30). Thus, the ubiquitin-based ED-B-binding molecules described in this paper show affinities and specificities that are in the upper range of ED-B-binding molecules published to date. In addition, selective immunohistochemical staining of vessel-associated ED-B in F9 tumor slices by ubiquitin-based binding molecules indicates high target specificity in complex tissues similar to the staining pattern obtained with L19 (31).

Ubiquitin-based binding molecules are small proteins of ∼17 kDa. Due to their small size, they are expected to undergo renal filtration in the glomerulus as the standard elimination pathway of small proteins sized below the filtration threshold (54). Thus, for such molecules, a similar short half-life as described for ubiquitin itself (55) can be expected. Indeed, a high clearance rate of 935 ml/h/kg was determined for dimeric ubiquitin variants.

Although a rapid systemic clearance is important for applications such as tumor imaging with tumor-targeting entities, a prolongation of the systemic circulation time is of interest for many other applications. In general, longer circulation provides the potential for prolonged and thereby increased tissue penetration mediating improved target occupancy. We have tested three standard approaches for half-life prolongation of biopharmaceuticals to modulate the molecular size of ubiquitin-based binding molecules and take advantage of the neonatal Fc receptor (FcRn)-mediated salvage pathway (*i.e.* Fc fusion, MSA fusion, and PEGylation) (56). Thus, molecules of 88, 85, and 60 kDa, respectively, were obtained. 77405-Fc is an N-terminal fusion of the 77405 variant to the constant part of human IgG1. Compared with 77405, this protein binds with about 4-fold increased affinity toward 67B89, most likely due to avidity effects (57). Linking the variant with an antibody Fc fragment results in increased molecular size exceeding the glomerular filtration threshold. In addition, interaction of the Fc fragment and the FcRn actively contributes to half-life extension because it enables intracellular recycling of Fc fragment-containing proteins, a pathway that ensures the long half-life of antibodies (58). Accordingly, Fc fusion of 77405 results in prolongation of the terminal half-life to 56 h. The obtained pharmacokinetic profile is comparable, for example, with a marketed Fc fragment-containing biopharmaceutical, Romiplostim, indicating that the Fc fusion of the 77405 variant prolonged the half-life within the expected range (59).

A second approach for half-life extension was attempted by fusing 77405 to serum albumin. For this approach, MSA was chosen over HSA because of the higher affinity of MSA to mouse FcRn compared with HSA (60). The MSA fusion protein is characterized by an about 2-fold increased *K_D_* value compared with 77405. The reduced affinity of 77405-MSA toward 67B89 might partially be related to its molecular size in conjunction with steric hindrance of target accessibility. Similar to the mechanism of half-life extension of the Fc fusion, the MSA fusion also benefits from reduced renal clearance as well as from the FcRn-mediated salvage pathway (61). Pharmacokinetics of the MSA fusion molecule is quite comparable with the pharmacokinetics of an Affibody-HSA (62) that possesses comparable molecular size. Thus, the general feasibility of half-life extension via MSA-fusion has been demonstrated, providing promising perspectives for Affilin serum albumin fusions for application in humans.

In a third approach, 77405 was PEGylated using a 40 kDa-branched PEG. Although PEGylation is a standard procedure utilized for many biopharmaceuticals (63) primarily aiming at preventing renal filtration, this approach is well known for steric interference of the PEG with target interaction (64). For example, for PEG-INF-α2a, a more than 10-fold reduced binding affinity compared with non-PEGylated INF is reported (65). We PEGylated variant 77405, yielding a protein with a molecular mass of 60 kDa. Although this molecule is smaller based on the nominal molecular weight compared with 77405-Fc and 77405-MSA, the large water binding capacity of PEG results in a hydrodynamic radius that easily exceeds the glomerular filtration threshold (44). 77405-PEG is characterized by an about 3-fold increased *K_D_* value compared with 77405, demonstrating only a minor decrease in affinity still sufficient to allow effective target binding and tumor targeting. In addition, the small influence of PEGylation on affinity reveals the compatibility of N-terminally conjugated PEG with preservation of binding activity of the binding molecule. The terminal half-life of 77405-PEG is determined to be 20 h and therefore quite comparable with the half-life of 77405-MSA. However, 77405-PEG is the conjugate with the slowest clearance of all three tested half-life extension approaches, which is in accordance with the inertness of PEG in biological systems. Overall, all three half-life prolongation approaches represent well suited technologies for increasing the blood residence time of ubiquitin-based binding molecules while only marginally interfering with target interaction.

Biodistribution of radiolabeled variant 77405 was investigated in the F9 teratocarcinoma mouse model. Independent of the labeling method, 77405 showed a tumor accumulation of between 6% and 7% ID/g at 2 h after administration but only weak tumor retention at 16 h postinjection. However, this is comparable with the tumor accumulation of the aforementioned Fynomer and L19 monomer, with 5.60% and 5.08% ID/g at 4 h after administration and 0.69% and 1.69% ID/g at 24 h after administration, respectively (28, 30). In addition, tumor/blood ratios of all three molecules are comparable as well.

Increasing the serum half-life of 77405 via Fc fusion and thereby generating a bivalent binding molecule clearly resulted in prolonged tumor retention comparable with that of L19 dimer (41). This effect is expected to be related to the increased molecular size of 77405-Fc together with its high affinity and avidity, leading to prolonged circulation and tumor retention. Similarly, a dimerized ED-B binding Fynomer showed prolonged tumor retention as well when compared with the monomeric protein (28). Overall, the data on tumor accumulation obtained with ubiquitin-based ED-B-binding molecules are expected to be within the range of other ED-B-binding proteins, thus reflecting the interrelationship of extracellular target expression, molecular size, and affinity as well as avidity of ED-B-binding proteins.

Ubiquitin qualifies as a well suited scaffold protein due to its superior biochemical and biophysical properties. Ubiquitin-based binding molecules are proteins with high stability, affinity, and specificity, as exemplified for the tumor-specific target ED-B. Tumor accumulation and retention of targeted therapeutics depend on a complex interplay of molecular parameters, such as affinity, size, half-life, extravasation propensity, and others. Interestingly, maximum affinity and *in vivo* half-life are not necessarily preferred to, for example, optimized tumor/blood ratios. Here we demonstrate the generation of specific ubiquitin-derived binding molecules with affinities spanning several logs down to the low picomolar range and half-life modulation from hours to days. Thus, the ubiquitin-based scaffold technology holds promising perspectives for therapeutic indications requiring specific product profiles. In addition, the ease of modular engineering of the ubiquitin-based scaffold technology enables the generation of multivalent and multispecific molecules with functionalities tailored to specific therapeutic applications that may complement current antibody formats.

## Footnotes

3 S. Lorey, E. Fiedler, A. Kunert, J. Nerkamp, C. Lange, M. Fiedler, E. Bosse-Doenecke, M. Meysing, M. Gloser, C. Rundfeldt, U. Rauchhaus, I. Hänssgen, T. Göttler, A. Steuernagel, U. Fiedler, and U. Haupts, unpublished data.

4 A. Katzschmann, manuscript in preparation.

## Footnotes

2 The abbreviations used are:

ED-B: extradomain B
6789: sequence of fibronectin domains 6, 7, 8, and 9
6789thc: 6789 protein containing a His tag and a C-terminal cysteine
67B89: ED-B domain B flanked by its neighboring N-terminal domains 6 and 7 and C-terminal domains 8 and 9
67B89thc: 67B89 protein containing a His tag and C-terminal cysteine
Fab: fragment antigen-binding
Fc: fragment crystallizable
HSA: human serum albumin
Nb67B89: N-terminal biotinylated 67B89
MSA: mouse serum albumin
HSA: human serum albumin
^125^I-SIB: *N*-succinimidyl 3-^125^I-benzoate
ID: injected dose
POD: peroxidase
SPR: surface plasmon resonance
*T_m_*: melting temperature
SUMO: small ubiquitin-like modifier
SE-HPLC: size exclusion HPLC
MALS: multiangle laser light scattering
BisTris: bis(2-hydroxyethyl)iminotris(hydroxymethyl)methane.
